# Evaluation of Pancreatic Steatosis in Patients with Celiac Disease

**DOI:** 10.5152/tjg.2025.25069

**Published:** 2025-10-20

**Authors:** Ahmet Tarhan, Yasemin Gökden, Sinem Aydemir, Suzan Önol, Fatma Zülal Özek

**Affiliations:** 1 Department of Internal Medicine, Health Sciences University Prof.Dr. Cemil Taşcıoğlu City Hospital, İstanbul, Türkiye; 2 Department of Gastroenterology, Health Sciences University Prof.Dr. Cemil Taşcıoğlu City Hospital, İstanbul, Türkiye; 3 Department of Radiology, Health Sciences University Prof.Dr. Cemil Taşcıoğlu City Hospital, İstanbul, Türkiye

**Keywords:** Celiac disease, gluten, hepatic steatosis, pancreatic diseases, ultrasonography

## Abstract

**Background/Aims:**

Celiac disease, a chronic autoimmune disorder, has been reported to be associated with pancreatic involvement, including exocrine pancreatic insufficiency, pancreatitis, and cystic fibrosis. The aim of this cross-sectional study was to determine the frequency of pancreatic steatosis (PS) in patients with celiac disease and compare it with that in healthy controls.

**Materials and Methods:**

Sixty patients with celiac disease and 60 healthy participants were included in this study. Biochemical and hematological parameters were collected from all participants. Hepatic steatosis (HS) and PS were diagnosed by ultrasonography and were compared between the groups.

**Results:**

Age, gender, and body mass index were similar between the groups (*P* > .05). Pancreatic steatosis (81.7%) and HS (66.7%) were more prevalent in the celiac group compared to healthy controls (*P* < .001). A positive and significant correlation was found between PS and HS in the celiac group (rho = 0.464, *P* < .05). Hepatic steatosis and PS did not differ between tissue transglutaminase–Immunoglobulin A (IgA)-positive and -negative patients with celiac disease (*P* > .05). No differences in HS or PS were found between celiac patients who adhered to a gluten-free diet and those who did not (*P* > .05).

**Conclusion:**

Celiac disease may be associated with an increased risk of HS and PS.

**Main Points**

- Celiac patients might be at an increased risk of both pancreatic steatosis (PS) and hepatic steatosis (HS).
- Celiac patients could be at risk for both PS and HS, regardless of their tissue transglutaminase–IgA status.
- It may be important to monitor celiac patients for PS when HS is present, to avoid possible complications associated with PS.

## Introduction

Celiac disease (CD) is an autoimmune disorder that triggers an immune response to gluten in individuals with a genetic predisposition.^1^ The etiology of CD involves a combination of genetic and environmental factors, as well as the role of the microbiome. This disease is significant as it can affect individuals across a wide age range and is associated with increased morbidity and mortality.^1^ The prevalence of CD has been reported to be approximately 1% globally, with an increasing prevalence worldwide.^1^ While the small intestine is primarily affected, CD has been reported to impact various organs and structures, including the liver, skin, and pancreas.^1^

The hepatic manifestations of CD have been extensively documented in the literature.^2^^,^^3^ Celiac disease has been identified as a potential cause of elevated hepatic enzyme levels^2^ and has also been associated with non-alcoholic fatty liver disease (NAFLD) and autoimmune liver disorders.^3^ Accordingly, it is recommended that patients presenting with hepatic steatosis (HS) or unexplained elevations in hepatic enzymes be evaluated for underlying CD.^4^

Both endocrine and exocrine pancreatic functions have been found to be negatively affected in CD. Celiac patients have been reported to be at risk for acute and chronic pancreatitis,^5^ possibly due to malnutrition, papillary stenosis, or immune mechanisms.^6^ Exocrine pancreatic insufficiency has also been reported in celiac patients, potentially linked to impaired enteric hormone activity.^6^^,^^7^ Additionally, increased risks of autoimmune pancreatitis and cystic fibrosis have been noted in patients with CD.^6^ However, the relationship between CD and pancreatic steatosis (PS), defined as fat infiltration of the pancreas, has not been previously investigated.^8^

Several mechanisms have been proposed to explain PS in CD. Chronic low-grade inflammation, which has been recognized as a hallmark of untreated CD, has been reported to promote ectopic fat accumulation via proinflammatory cytokines such as interleukin-6.^9^ Additionally, intestinal malabsorption, dysregulated enteric hormones, and microbiota-derived metabolites have been suggested to alter lipid metabolism, leading to fat deposition in non-adipose tissues such as the pancreas.^10^^,^^11^ Increased intestinal permeability has also been proposed to impair gut-liver-pancreas signaling, allowing endotoxins to reach the pancreas and trigger steatosis.^11^

Since CD has been found to be associated with PS^6^ and HS^2^^,^^3^ and a positive correlation between PS and HS has also been described,^12^ the hypothesis of the current study is that there is an increased frequency of PS in patients with CD. Therefore, the aim of this study was to characterize the frequency of PS in patients with CD and compare it to controls.

## Materials and Methods

### Study Groups

For this cross-sectional study, a total of 120 participants between the ages of 18 and 65 were equally divided into 2 groups: the CD group and the healthy controls. The celiac group consisted of patients with a confirmed diagnosis of CD, established through serologic testing (tissue transglutaminase–IgA [TTG-IgA] antibody positivity), endoscopic findings (the presence of scalloped duodenal folds, grooves, and fissurations), and histologic assessment (Marsh classification). The healthy group consisted of participants who presented for routine check-ups at the Department of Internal Medicine, Health Sciences University Prof.Dr. Cemil Taşcıoğlu City Hospital, İstanbul, Türkiye between May 2022 and September 2022. Ethical committee approval was obtained from Health Sciences University Prof. Dr. Cemil Taşcıoğlu City Hospital, İstanbul, Türkiye (approval number: E-48670771-514.99) on May 9, 2022, and the current study was conducted in full accordance with the Declaration of Helsinki (revised in 2013). Written informed consent was obtained from all participants.

The exclusion criteria for all participants were as follows: pregnancy and lactation, a history of cancer and undergoing chemotherapy and radiotherapy, chronic renal failure, alcoholic hepatitis, alcohol consumption (ethanol intake >28 g/day for males, >14 g/day for females),^13^ liver cirrhosis, and a history of pancreatitis. For the healthy group, the presence of CD, hepatic and pancreatic pathologies, additional systemic diseases, and a history of chronic medication use, TTG-IgA levels higher than the normal reference value (<20 RU/mL) were considered exclusion criteria. To exclude patients with isolated IgA deficiency, total serum IgA levels were measured in all participants. Individuals with confirmed IgA deficiency were not included in the study.

### Data Collection

Data on age, body mass index, waist circumference, smoking and alcohol consumption, hypertension, and diabetes mellitus were collected from all participants. Additionally, total cholesterol, Low-Density Lipoprotein (LDL) cholesterol (LDL-C), High-Density Lipoprotein (HDL) cholesterol (HDL-C), triglycerides, urea, creatinine, aspartate aminotransferase, alanine aminotransferase, alkaline phosphatase, gamma glutamyl transferase, lactate dehydrogenase, white blood cell, hemoglobin, hematocrit, platelet, amylase, lipase, fasting blood sugar, insulin levels, and Homeostatic Model Assessment of Insulin Resistance (HOMA-IR) values were also determined from blood samples of all participants. Metabolic syndrome was determined by the presence of 3 or more of the criteria established by the National Cholesterol Education Program/Adult Treatment Panel III.^14^ It has been suggested to define ethnic classifications for the waist circumference,^15^ and a waist circumference of ≥100 cm in males and ≥90 cm in females has been accepted as the criterion for abdominal obesity in the Turkish population.^16^

In the celiac group, initial symptoms of the disease, tissue transglutaminase (TTG)-IgA antibody levels, illness duration, age of diagnosis, presence of additional diseases, and anti–thyroid peroxidase positivity were recorded. Patients were also classified according to the TTG-IgA levels determined within the last month.^17^ Body mass index (BMI) was recorded for all patients based on World Health Organization criteria. Body mass index <18.5 kg/m^2^ was classified as underweight, 18.5-24.9 kg/m^2^ as normal weight, 25-29.9 kg/m^2^ as overweight, and ≥30 kg/m^2^ as obese.^18^ The adherence of the patients to the gluten-free diet was also assessed and classified into 4 different categories. The patients were offered 4 options for dietary compliance, which were classified as a) perfectly compliant with the diet, b) usually adherent to the diet, c) occasionally adherent to the diet, and d) not at all. Ferritin, iron, total iron binding capacity, transferrin saturation, vitamin B12, folate, and 25(OH)D3 levels of the patients in the celiac group were also recorded.

### Ultrasonography

**Figure 1.**
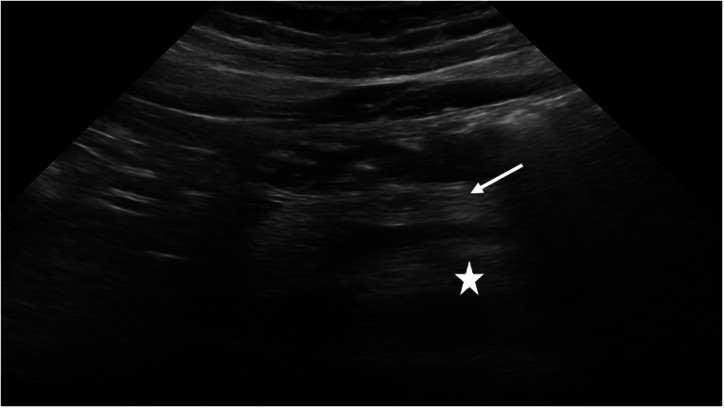
A representative image of pancreatic echogenicity in abdominal ultrasound. Grade 3: pancreatic echogenicity (thin arrow) is similar to or higher than the retroperitoneal fat (asterisk).

All ultrasonographic evaluations were performed by a single radiologist with 22 years of experience in abdominal imaging. Intra-examiner reliability was evaluated through repeated assessments of selected ultrasound images and was found to exceed 98%, indicating excellent consistency. While monitoring pancreatic echogenicity using ultrasound, the head and neck of the pancreas were indirectly compared with the right kidney, utilizing 2 parallel ultrasound windows (Figure 1).^19^

For the classification of PS, a 3-grade staging system based on subjective visual assessment was used as follows: Grade 0: pancreas and renal echogenicity are similar; Grade 1: pancreas echogenicity is slightly higher than renal echogenicity; Grade 2: pancreas echogenicity substantially higher than renal echogenicity but lower than retroperitoneal fat echogenicity; Grade 3: pancreas echogenicity is similar to or higher than that of the retroperitoneal fat echogenicity.^8^ For the classification of HS, a subjective visual staging scale was also used as follows: Grade 0: hepatic echogenicity equal to or slightly greater than that of the renal cortex and spleen;^20^ Grade 1: diffusely increased hepatic echogenicity (greater than renal cortex and spleen) but periportal and diaphragmatic echogenicity is still appreciable; Grade 2: diffusely increased hepatic echogenicity obscuring periportal echogenicity but diaphragmatic echogenicity is still appreciable; Grade 3: diffusely increased hepatic echogenicity obscuring periportal as well as diaphragmatic echogenicity.^21^

### Statistical Analysis

All statistical analyses were performed using software (IBM SPSS Corp.; Armonk, NY, USA). Numerical data are presented as mean ± SD, and categorical data are presented as counts and percentages. The normal distribution of the data was assessed using the Kolmogorov–Smirnov test. The differences between groups were determined using parametric tests (independent samples *t*-test) for normally distributed data and non-parametric tests (Mann–Whitney *U*) for non-normally distributed data. Pearson’s chi-squared test (with Yates’ Correction) was used for the comparison of categorical data. Correlation analysis was performed using Spearman and Pearson correlation analysis. Binary logistic regression analysis was conducted to identify independent risk factors. *P* values <.05 were considered statistically significant.

A power analysis could not be conducted at the beginning of the study due to the absence of a similar study in the same field. Therefore, a post hoc power analysis was performed at the end of the study based on the PS data, which indicated a power of 93.8% with a total of 120 participants (Effect size 0.395, α = 0.05, df = 5, Noncentrality parameter λ = 18.80, Critical χ^2^ = 11.07).

## Results

### Demographic, Biochemical, and Hematological Characteristics

At the beginning of the study, 10 patients with diabetes mellitus, 2 patients with a history of pancreatitis, 1 lactating patient, 3 patients with chronic renal failure, and 1 patient with a history of high alcohol consumption were excluded from the celiac group. A total of 120 patients were included in this study, with 60 celiac patients (37 females and 23 males) and 60 healthy controls (35 females and 25 males). Age and gender, BMI, waist circumference, smoking, and alcohol consumption were similar between the groups (Table 1) (*P* > .05). The hematological and biochemical values were within the normal reference range in the groups (Table 1) (*P* > 0.05).

Further subgroup analysis was conducted in patients with CD. The age at diagnosis of CD patients was 30.12 ± 12.83 years. The time period from diagnosis (illness duration) was 8.07 ± 9.35 years, and for 5 participants, the illness duration was less than 1 year. Adherence to a gluten-free diet was also questioned. Out of 60 participants, 27 strictly followed the diet (45%), 28 mostly adhered to it (46.7%), 2 occasionally complied (3.3%), and 3 were not compliant (5%). Twenty-eight patients tested positive for TTG-IgA (≥20 RU/mL), while 32 tested negative (<20 RU/mL). Anti–thyroid peroxidase positivity was positive (>9 IU/mL) in 13 patients and negative in 47 patients, falling below the reference range. Patients in the celiac group were evaluated for levels of ferritin, iron, total iron binding capacity, transferrin saturation, vitamin B12, folate, and 25(OH)D3 levels, which were all within the normal reference range.

At the time of diagnosis, initial duodenal biopsies were available for all 60 patients with CD, showing Marsh classification as follows: 4 patients (6.7%) were classified as type 1, 6 (10.0%) as type 2, 18 (30.0%) as type 3a, 26 (43.3%) as type 3b, and 6 (10.0%) as type 3c. All patients were advised to follow a gluten-free diet. During follow-up, control biopsies were performed in 41 patients due to either persistent serological positivity or ongoing complaints despite negative serology. Among these, Marsh classification revealed 12 (29.3%) as type 0, 4 (9.8%) as type 1, 16 (39.0%) as type 2, 8 (19.5%) as type 3a, and 1 (2.4%) as type 3b. During the follow-up period, all patients underwent a single abdominal ultrasound to assess PS, and serological activity was evaluated based on TTG-IgA levels measured immediately before the scan.

### Radiological Findings

Due to the limited number of participants in the subgroups, all participants were categorized into 2 main groups for both PS and HS evaluations: Grade 0 and Grade 1-3. The detailed distribution of PS and HS grades across groups is provided in Table 2. Significant differences in PS and HS were noted between the celiac and healthy control groups (*P* < .001) (Table 2). Hepatic steatosis and PS were also compared in the CD patients according to their TTG-IgA values. Neither HS nor PS exhibited statistically significant differences between TTG-IgA positive or negative patients with CD (*P* > .05) (Table 3).

A correlation analysis was conducted to examine the possible relationship between HS and PS. In both groups, a positive correlation was observed between HS and PS severity grades. In celiac patients, a strong and statistically significant positive correlation was found between both HS and PS with age and waist circumference (*P* < .001) (Table 4). In addition, in celiac patients, a positive and significant correlation was found between HS and LDL-C, total cholesterol, and triglycerides (*P* < .01). The same parameters were also positively and significantly correlated with PS in patients with CD (*P* < .01) (Table 4).

The risk factors affecting HS and PS were further evaluated using binary logistic regression analysis in terms of univariate and multiple models. Univariate analysis revealed that as age increases, the risk of HS increased by a factor of 1.155 (OR 95% CI, 1.073 to 1.243; *P* < .001). Similarly, an increase in waist circumference also raises the risk of HS by 1.083 times (OR 95% CI, 1.018 to 1.153; *P* = .012). However, in the multiple model analysis, while an increase in age elevates the risk of HS by 1.161 times (OR 95% CI, 1.057 to 1.275; *P* = .002), waist circumference does not significantly affect HS risk (*P* = .855). On the other hand, the univariate analysis showed that as age increases, the risk of PS rises by a factor of 1.159 (OR 95% CI, 1.056 to 1.272; *P* = .002). Similarly, an increase in waist circumference contributes to a 1.236-fold increase in PS risk (OR 95% CI, 1.089 to 1.403; *P* = .001). However, in the multiple model analysis, age does not significantly impact PS risk (*P* = .105). Instead, an increase in waist circumference raises the risk of PS by a factor of 1.182 (OR 95% CI, 1.029 to 1.357; *P* = .018).

## Discussion

Celiac disease has been associated with impaired pancreatic function, including exocrine pancreatic insufficiency, acute or chronic pancreatitis, and cystic fibrosis.^6^ The current findings of this study revealed that both HS and PS were significantly more common in celiac patients than in healthy controls. A strong and positive correlation was observed between HS and PS, as well as between these conditions and metabolic parameters such as waist circumference, total cholesterol, LDL-C, and triglyceride levels. Neither TTG-IgA positivity nor dietary compliance significantly affected the prevalence or severity of HS or PS. Age and waist circumference were significant independent risk factors for HS and PS, respectively. These results suggest that CD may be associated with increased risk of PS and HS.

Recently, in the literature, PS has been reported to be highly prevalent, with a prevalence of 68% in Türkiye.^22^ The literature also indicates that individuals with fatty pancreas have a higher risk of developing diabetes.^23^ Additionally, individuals with newly diagnosed type 2 diabetes have been found to have significantly higher pancreatic fat content.^24^ Pancreatic fatty infiltration exceeding 25% has been found to be associated with an increased risk of developing type 2 diabetes.^24^ In addition, CD antibody positivity has also been reported at the initial presentation of type 1 diabetes.^25^ In the current study, FBS levels were found to be significantly higher in CD patients compared to healthy controls, although the levels in both groups were within the reference values (*P* < .05). Consequently, the current findings necessitate attention and further evaluation regarding the development of diabetes risk in patients with CD.

In the current study, a comprehensive analysis of metabolic parameters demonstrated significant associations between both HS and PS with several key metabolic indicators. Specifically, HS and PS were positively correlated with waist circumference, total cholesterol, LDL-C, and triglyceride levels, particularly among patients with CD (*P* < .01). These findings align with existing literature indicating that ectopic fat accumulation in non-adipose tissues such as the liver and pancreas is frequently linked to central obesity and dyslipidemia.^26^ Interestingly, despite the well-established role of insulin resistance in the pathophysiology of metabolic syndrome, no significant correlations were found between HS or PS and insulin levels or HOMA-IR in either group. This observation suggests that pancreatic fat deposition may, in some cases, develop independently of insulin resistance.^27^^,^^28^ Alternative mechanisms such as chronic low-grade inflammation, impaired intestinal barrier function, or alterations in gut microbiota have been proposed as contributing factors, especially in CD.^29^ The present results highlight the complexity of ectopic fat accumulation and emphasize the need for broader metabolic monitoring in celiac patients, including lipid profile and abdominal fat distribution, even when glycemic indices appear within normal limits.

The association between CD and NAFLD has been examined in the literature. In a study by Tovoli et al,^30^ NAFLD was diagnosed in 34.7% of CD patients adhering to a gluten-free diet (defined by negative TTG-IgA, absence of gluten intake for 6 months, and no symptoms). A 3-fold higher risk of NAFLD was also reported in CD patients compared to healthy individuals.^30^ In the present study, HS was detected in 66.7% of CD patients and in 33.3% of healthy controls. Consistent with previous findings, no significant differences in HS prevalence were observed between diet-compliant and non-compliant CD patients. These findings suggest that an increased risk of HS may be associated with CD, regardless of dietary adherence. A cohort study by Reilly et al,^31^ involving 26 816 celiac patients, examined the relationship between CD and NAFLD. The study reported an increased risk of NAFLD in celiac patients compared to the healthy population. The highest risk occurred within the first year after celiac diagnosis but continued to persist for up to 15 years after diagnosis. Similarly, in the current study, the incidence of HS was significantly higher in the celiac group compared to the healthy control group. The duration of illness was 8.07±9.35 years, but it was not correlated with HS or PS. This lack of correlation could be attributed to the differences in study designs and sample sizes between the 2 studies.

Ciccone et al^32^ evaluated the HS risk after following a gluten-free diet in celiac patients and reported that HS was detected as 1.7% at the time of celiac diagnosis but increased to 11.1% following a gluten-free diet. Imperatore et al^33^ found HS in 25.9% of celiac patients at the time of diagnosis, while the incidence rose to 37.5% after following a gluten-free diet for 1 year. Both studies emphasized the importance of informing celiac patients about the increased risk of HS when on a gluten-free diet. Celiac patients following a gluten-free diet have been observed to consume a high percentage of simple sugars and saturated fats, with fewer fiber-rich foods and complex carbohydrates, leading to imbalanced nutrition.^34^ This type of diet is believed to increase the risk of NAFLD.^30^^,^^31^ However, in this study, no significant difference was found in terms of HS and PS grades between patients who strictly adhered to a gluten-free diet and those who did not. Therefore, even among patients who are compliant with their diet, the degree of steatosis may still increase. Recent studies have shown that certain conditions persist in celiac patients despite a lifelong gluten-free diet, including increased intestinal permeability, small bowel growth, microbiota changes, exocrine pancreatic insufficiency, and low-grade gastrointestinal inflammation. Some of these factors are believed to be responsible for the development of NAFLD.^34^

The association between HS and PS has been evaluated in the literature. Sezgin et al^22^ reported a significant relationship between the severity of PS and the presence of HS. They stated that the prevalence of HS was 57% in patients with mild PS, 74% in those with moderate PS, and 90% in patients with severe PS. Al-Haddad et al^35^ reported a 14-fold increased risk of hyperechogenic pancreas in individuals with HS. Van Geenen et al^36^ found a positive correlation between pancreatic and hepatic fatty infiltration. Patel et al^37^ stated that fatty pancreas was associated with HS in individuals with NAFLD. Wang et al^23^ detected that 67.2% of study participants with fatty pancreas had NAFLD. In this current study, a positive and statistically significant correlation between HS and PS was found when examining both the celiac group and all participants. Therefore, it may be stated that there is a positive association between PS and HS in individuals with CD.

Since pancreatic echogenicity may be influenced by acute or chronic pancreatitis, all participants were monitored for pancreatic enzyme elevations to minimize the impact of potential confounding factors contributing to pancreatic fat accumulation. No significant differences in enzyme levels were observed between the study groups. Similarly, no differences were found in factors previously identified as potential risk factors for PS, including BMI, significant alcohol consumption,^35^ and age.^38^ Therefore, the observed increase in pancreatic echogenicity may be attributed to celiac disease.

This study has some limitations. Firstly, in the current study PS was not confirmed histologically or by MRI. However, ultrasonography, a reliable and non-invasive method that has been widely used in studies evaluating PS,^23^^,^^39^ was employed in the current study. While all ultrasound evaluations were performed by an experienced radiologist using standardized protocols, the absence of histologic validation remains a methodological limitation. Secondly, the relatively small sample size may limit the generalizability of the findings. In addition, follow-up biopsy data were not available for all patients, which prevented a comprehensive assessment of histologic changes over time and restricted the ability to evaluate the relationship between Marsh classification and PS in the entire study cohort. Histologic remission could not be evaluated in patients who were asymptomatic, strictly adherent to a gluten-free diet, and seronegative at the time of follow-up, as control biopsies were not performed in this subgroup. Therefore, the association between PS and histologic remission could not be assessed in these patients.

In the current study, both HS and PS were significantly more frequent in patients with CD compared to healthy controls. Among celiac patients, a positive and statistically significant correlation was observed between HS and PS. These findings suggest that individuals with CD may be at increased risk for PS and HS.

## Figures and Tables

**Table 1.** Demographic and Laboratory Characteristics of Study Participants

	CD	Healthy Controls	Reference Value
Female/Male	37/23	35/25	
Age	38.25 ± 11.89	34.82 ± 9.24	
BMI	23.86 ± 4.11	23.66 ± 2.44	
Waist circumference (cm)	81.23 ± 10.41	83.93 ± 11.48	
Smoking (Yes/No)	17/43	16/44	
Alcohol consumption (Yes/No)	0/60	0/60	
Total cholesterol	170.68 ± 38.28	178.30 ± 38.57	<200 mg/dL
LDL-C	103.07 ± 31.56	106.09 ± 34.07	<100 mg/dL
HDL-C	51.54 ± 14.23	52.42 ± 13.61	>55 mg/dL no risk 35-55 moderate risk <35 high risk
Triglyceride	80.88 ± 48.19	96.98 ± 53.70	<200 mg/dL
Urea	23.65 ± 8.60	25.100 ± 7.23	16.6-48.5 mg/dL
Creatinine*	0.70 ± 0.13	0.78 ± 0.17	0.70-1.20 mg/dL
AST	19.81 ± 6.49	17.85 ± 4.65	0-40 U/L
ALT	19.19 ± 9.59	17.98 ± 11.24	0-41 U/L
ALP	69.10 ± 23.32	64.02 ± 21.81	40-129 U/L
GGT	15.03 ± 12.01	16.20 ± 9.50	10-71 U/L
LDH	173.85 ± 21.20	177.74 ± 32.79	135-225 U/L
WBC	6.29 ± 1.47	6.91 ± 1.79	3.8-10 /uL
HGB	13.51 ± 1.87	14.04 ± 1.57	11.7-16 g/L
HCT	40.82 ± 5.49	41.14 ± 4.14	35%-47%
PLT	259 383.33 ± 61 828.93	253 800.34 ± 62 661.51	150-400 /uL
Amylase	69.35 ± 30.44	66.43 ± 23.32	28-100 U/L
Lipase	27.67 ± 8.74	30.63 ± 9.51	13-60 U/L
FBS*	91.02 ± 10.48	85.46 ± 8.00	74-100 mg/dL
Insulin*	7.77 ± 4.79	9.00 ± 3.77	2.6-24.9 mU/L
HOMA-IR	1.81 ± 1.91	1.30 ± 0.89	≥2.5 significant
Metabolic syndrome (Yes/No)*	2/58	0/60	≥3/6 criteria

Continuous variables are expressed as median (interquartile range). Categorical variables are reported as frequencies (%).

ALP, alkaline phosphatase; ALT, alanine aminotransferase; AST, aspartate aminotransferase; BMI, body mass index; CD, celiac disease; FBS, fasting blood sugar; GGT, gamma glutamyl transferase; HCT, hematocrit; HDL-C, HDL cholesterol; HGB, hemoglobin; HOMA-IR, Homeostatic Model Assessment insulin Resistance; LDH, lactate dehydrogenase; LDL-C, LDL cholesterol; PLT, platelet; WBC, white blood cell.

*P < .05, statistically different between groups.

**Table 2.** Comparison of Hepatic Steatosis and Pancreatic Steatosis in Participants of Both Study Groups

		CD [n (%)]	Healthy Controls [n (%)]	*P*
Hepatic steatosis	Grade 0	20 (33.3)	38 (63.3)	**.000**
	Grade 1-3 Grade 1 Grade 2 Grade 3	40 (66.7) 36 (60) 4 (6.7) 0 (0)	22 (36.7) 16 (26.7) 5 (8.3) 1 (1.7)	
Pancreatic steatosis	Grade 0	11 (18.3)	34 (56.7)	**.000**
	Grade 1-3 Grade 1 Grade 2 Grade 3	49 (81.7) 15 (25) 15 (25) 19 (31.7)	26 (43.3) 15 (25) 9 (15) 2 (3.3)	

CD, celiac disease.

**Table 3.** Comparison of Hepatic Steatosis and Pancreatic Steatosis in Celiac Group According to Tissue Transglutaminase–IgA

		TTG-IgA Negativity n:32 [n (%)]	TTG-IgA Positivity n:28 [n (%)]	*P*
Hepatic steatosis	Grade 0	11 (34.4)	9 (32.1)	>.05
	Grade 1-3 Grade 1 Grade 2 Grade 3	21 (65.6) 19 (52.8) 2 (6.3) 0 (0)	19 (67.9) 17 (60.7) 2 (7.1) 0 (0)	
Pancreatic steatosis	Grade 0	5 (15.6)	6 (21.4)	>.05
	Grade 1-3 Grade 1 Grade 2 Grade 3	27 (84.4) 8 (25) 8 (25) 11 (34.4)	22 (78.6) 7 (25) 7 (25) 8 (28.6)	

TTG-IgA, tissue transglutaminase–IgA-.

**Table 4.** Correlation Analysis of Hepatic Steatosis and Pancreatic Steatosis in the Study Participants

	CD		Healthy Controls		All Participants	
	Rho	*P*	Rho	*P*	Rho	*P*
HS and PS	0.464	.000*	0.377	.003*	0.496	.000*
HS and Age	0.604	.000*	0.229	.079	0.434	.000*
PS and Age	0.687	.000*	−0.001	.997	0.432	.007*
HS and Gender	−0.274	.183	0.177	.175	0.011	.906
PS and Gender	−0.019	.883	0.143	.276	0.007	.940
HS and duration of disease	0.194	.137	–	–	–	–
PS and duration of disease	0.193	.139	–	–	–	–
HS and TTG-IgA level	−0.050	.677	–	–	–	–
PS ve TTG-IgA level	−0.095	.280	–	–	–	–
HS and BMI	−0.032	.807	0.262	.062	0.086	.369
PS and BMI	0.190	.147	0.137	.334	0.144	.130
HS and Waist circumference	0.424	.001*	0.508	.000*	0.414	.000*
PS and Waist circumference	0.625	.000*	0.384	.002*	0.406	.000*
HS and Total Cholesterol	0.391	.002*	0.2236	.070	0.277	.002*
HS and LDL-C	0.344	.007*	0.283	.029*	0.289	.001*
HS and HDL-C	0.013	.921	−0.380	.003*	−0.201	.028*
HS and Triglyceride	0.494	.000*	0.393	.002*	0.366	.000*
HS and FBS	−0.029	.827	0.109	.471	0.108	.271
HS and Insulin	−0.053	.689	−0.004	.980	−0.115	.240
HS and HOMA-IR	−0.076	.562	0.004	.981	−0.086	.341
PS and Total Cholesterol	0.286	.002*	0.105	.426	0.192	.036*
PS and LDL-C	0.498	.000*	0.170	.193	0.293	.001*
PS and HDL-C	−0.225	.084	−0.224	.085	−0.203	.026*
PS and Triglyceride	0.480	.000*	0.100	.450	0.366	.000*
PS and FBS	0.000	.997	0.260	.081	0.212	.030*
PS and Insulin	0.189	.148	−0.006	.969	−0.032	.746
PS and HOMA-IR	0.166	.206	0.064	.674	0.031	.750

BMI, body mass index; CD, celiac disease; FBS, fasting blood sugar; HDL-C, HDL cholesterol; HOMA-IR, Homeostatic Model Assessment Insulin Resistance; HS, hepatic steatosis; LDL-C, LDL cholesterol; PS, pancreatic steatosis; TTG-IgA, tissue transglutaminase–IgA.

*P < .05, statistically different between groups.

## Data Availability

The data that support the findings of this study are available on request from the corresponding author.
